# Androgenic-anabolic steroids use among bodybuilders in western Iran: application of ridge logistic regression model

**DOI:** 10.1186/s13102-023-00616-4

**Published:** 2023-01-11

**Authors:** Sanaz Khalili, Sahar Khoshravesh, Majid Barati, Hossein Mahjoub, Javad Faradmal

**Affiliations:** 1 Department of Biostatistics School of Public Health, Hamadan University of Medical Sciences, Hamadan, Iran; 2 Social Determinants of Health Research Center, Hamadan University of Medical Sciences, Hamadan, Iran; 3 Autism Spectrum Disorders Research Center, Hamadan University of Medical Sciences, Hamadan, Iran; 4 Department of Biostatistics School of Public Health, Modeling of Noncommunicable Diseases Research Center, Hamadan University of Medical Sciences, Hamadan, Iran

**Keywords:** Bodybuilder, Steroid, Collinearity, Ridge logistic regression model

## Abstract

**Introduction:**

Nowadays, the use of androgenic-anabolic steroids (AAS) by competitive and non-competitive bodybuilders and its side effects have become a major public health problem. Many studies have focused on determining the role and severity of various factors in AAS use, but the existence of collinearity between the factors leads to the non-significance of important factors. The study aimed to determine factors affecting the androgenic-anabolic steroids use in Iranian bodybuilders.

**Method:**

This descriptive-analytical study was performed on 280 male bodybuilders (142 non-competitive and 138 competitive bodybuilders) in Hamadan, west of Iran, in 2016. The participations were recruited a multistage sampling method. A self-administrated questionnaire was used, which included parts such as intrapersonal, interpersonal, and behavioral factors affecting on AAS use. To solve the consequences of collinearity was used ridge logistic regression model (RLRM) in R.3.5.1 software.

**Results:**

The mean age of bodybuilders was 25.21 years (SD = 6.31). The prevalence rate of AAS use among non-competitive and competitive bodybuilders was 27.5% and 34.1%, respectively. Factors such as age, time of starting bodybuilding, attitude, physical self-concept, behavioral intention, coach and friend use AAS, alcohol consumption, and supplement use were associated with AAS use among non-competitive and competitive bodybuilders.

**Conclusion:**

The results of the study indicated that a combination of intrapersonal, interpersonal and behavioral factors was effective on the androgenic-anabolic steroids use among Iranian bodybuilders. Adequate education about the side effects of AAS and improvement of individual skills seem to be helpful in reducing AAS use.

**Supplementary Information:**

The online version contains supplementary material available at 10.1186/s13102-023-00616-4.

## Introduction

The widespread use of muscular men in media has challenged the body image of men. Developments in bodybuilding, promotion in bodybuilding contests, and financial rewards in these contests can be very attractive for young men. Such issues have caused high interest in men toward this area, whether for fitness and gaining muscle or preparing for bodybuilding competitions. Thus, some competitive or non-competitive bodybuilders use androgenic-anabolic steroids (AAS) to speed up the muscle-building process [1–4].

Androgenic-anabolic steroids are classified under different trade names which are synthetic derivatives of the male hormone testosterone. AAS Abuse has become an important public health concern worldwide due to its serious and dangerous side effects. The evidence indicates that infertility, hair loss, pimples, acne, thickening of the voice, heart disease, cancer, and testicular atrophy are reported as the side effects of AAS abuse [5–8]. The AAS abuse has also led to psychological problems like depression, aggression, anxiety, and sleep disorders [7, 9–11]. The results of a meta-analysis (2014) reported that the worldwide prevalence rates of AAS use by athletes and recreational athletes were estimated at 13.4% and 18.4%, respectively [12]. The prevalence rate of AAS use by Iranian bodybuilders has been estimated 32.9%. [13].

The role of intrapersonal, interpersonal, and behavioral factors affecting the androgenic-anabolic steroids use has been documented among bodybuilders [14, 15]. In previous studies, a point that has not been paid attention to is that modeling these factors may have inherent collinearity. Collinearity is defined as the relationship between predictor variables that it has some serious effects such as inflation in the variance of estimators and the non-significance of important variables. On the other hand, the contribution of each predictor variable is not separable due to collinearity [16].

Due to the non-significance of the predictor variables arising from collinearity, some researchers fit a regression model for each predictor variable. Although this seems to eliminate the collinearity, it leads to model underestimation [17]. Because of the inherent collinearity between intrapersonal, interpersonal, and behavioral factors, it is essential to control the consequences of collinearity. In this study, factors affecting the use of anabolic steroids among bodybuilders in Hamadan were determined with an appropriate model.

## Methods

### Study setting, population, and sampling method

This descriptive-analytical study was performed on 280 male bodybuilders in Hamadan, west of Iran, in 2016. The participations were recruited a multistage sampling method. For this, each geographical region of Hamadan city was considered as a region (three regions). Then, 12 gyms were randomly chosen from each region and from each gym, 25 bodybuilders were selected by simple sampling method. In totally, 300 bodybuilders who 280 bodybuilders completed a questionnaire (Response rate = 93.3%). Inclusion criteria were considered ages > 15 years, attending gyms at least twice a week, and no history of physical or mental disorders. Before filling out the questionnaire, an informed consent form was obtained from bodybuilders. The questionnaires with incomplete information were excluded. In this study, a competitive and non-competitive bodybuilder were defined as a person who participates in bodybuilding competitions and a person who does not participate in any bodybuilding competition, respectively. The study was approved by the Ethics committee of Hamadan University of Medical Sciences, Iran (No. IR.UMSHA.REC.1395.356).

### Measurement

A self-administrated questionnaire was used. Detailed information is provided in the Additional file 1.

### Statistical methods and software

In this study the collinearity between predictor variables was examined by extracting the condition number. Because the condition number is based on the eigenvalues of the correlation matrix, it gives an accurate view of the collinearity status between the predictor variables. This index is the square root of the maximum eigenvalue divided by the minimum eigenvalue of the correlation matrix. This value was 198, which indicates severe collinearity [18]. In this survey, the response variable was AAS use (yes/no). The ridge logistic regression model (RLRM) was used to solve the consequences of collinearity, such as inflation, in the variance of estimators and non-significance of important variables [19]. Indeed, the RLRM is an extension of the ordinary logistic model. In RLRM, the variance of the estimator remarkably decreases by adding a small bias. Here, all the analyses were conducted with R.3.5.1 software at the significance level of 0.05.

## Results

Totally, 142 non-competitive bodybuilders and 138 competitive bodybuilders (113 people at the regional level and 25 people at the national level) participated in this study. Characteristics of participated bodybuilders is reported in Table 1. The mean age of bodybuilders was 25.21 years (SD = 6.31).

**Table 1:** Characteristics of participated bodybuilders (n = 280)

Variable	Non-competitive (n = 142)	Competitive (n = 138)
	n (%)	n (%)
*Material status*		
Single	116 (81.7)	107 (77.5)
Married	26 (18.3)	31 (22.5)
*Education level*		
Under diploma	17 (12.0)	16 (11.6)
Diploma	63 (43.7)	57 (41.3)
Academic	63 (44.3)	55 (47.1)
*Occupation*		
Employed	25 (17.6)	18 (13.1)
Self-employed	81 (57.0)	82 (59.4)
Unemployed	36 (25.4)	38 (27.5)
*AAS use*		
Yes	39 (27.5)	47 (34.1)
No	103 (72.5)	91 (65.9)

The mean (SD) ages of non-competitive and competitive bodybuilders were 24.88(6.49) and 25.54 (6.21), respectively. The prevalence rates of AAS use in non-competitive and competitive bodybuilders were 27.5% and 34.1%, respectively. Also, the relation between AAS use and bodybuilders' status (competitive and non-competitive) were tested with chi-square (*p* = 0.232).

Table 2 shows the results of affecting factors on AAS use for non-competitive bodybuilders. These results showed that people who are older are less likely to use AAS (OR = 0.881 [0.856–0.908]; *p* < 0.001). Also, subjects with more gym history are less likely to use AAS (OR = 0.902 [0.899–0.906]; *p* < 0.001). BMI and physical self-concept are other factors that have a negative relationship with AAS use. Bodybuilders with more BMI is less likely to use AAS (OR = 0.937 [0.896–0.980]; *p* = 0.005). This result is also true for physical self-concept (OR = 0.804 [0.776–0.835]; *p* < 0.001). On the other hand, the odds of AAS use increase by 16% in people who have a more positive attitude (OR = 1.164 [1.117–1.213]; *p* < 0.001).

**Table 2:** Affecting factors on AAS use for non-competitive bodybuilders

Variable	$\widehat{\upbeta }$	SE ($\widehat{\upbeta }$)	Odds ratio	95% CI	*p*-value
*Intrapersonal factors*					
Age	− 0.126	0.015	0.881	(0.856, 0.908)	< 0.001
Time of starting the bodybuilding	− 0.103	0.002	0.902	(0.899, 0.906)	< 0.001
Body mass index	− 0.065	0.023	0.937	(0.896, 0.980)	0.005
Attitude	0.152	0.021	1.164	(1.117, 1.213)	< 0.001
Subjective norms	0.011	0.027	1.011	(0.959, 1.066)	0.684
Physical self-concept	− 0.217	0.019	0.804	(0.776, 0.835)	< 0.001
Behavioral intention	0.174	0.028	1.190	(1.127, 1.257)	< 0.001
Behavioral willingness	0.033	0.033	1.034	(0.969, 1.103)	0.314
*Interpersonal factors*					
Coach use AAS	0.714	0.137	2.012	(1.561, 2.671)	< 0.001
Friend use AAS	0.847	0.128	2.332	(1.815, 2.998)	< 0.001
*Behavioral factors*					
Alcohol consumption	0.603	0.165	1.827	(1.323, 2.525)	< 0.001
Smoking	0.751	0.209	2.120	(1.407, 3.192)	< 0.001
Supplement use	0.532	0.133	1.702	(1.312, 2.209)	< 0.001

Bodybuilders with higher behavioral intentions have 19% higher odds of taking AAS (OR = 1.190 [1.127–1.257]; *p* < 0.001). When the coach is an AAS user, the odds of taking AAS for bodybuilders are 2 times (OR = 2.012 [1.561–2.671]; *p* < 0.001). In addition, for bodybuilders whose friends take AAS, the odds of taking AAS increase by 2.3 times (OR = 2.332 [1.815–2.998]; *p* < 0.001). Consumption of alcohol and supplements by bodybuilders increases the chance of steroid use by 82% and 70% (OR = 1.827 [1.323–2.525]; *p* < 0.001, OR = 1.702 [1.312–2.209]; *p* < 0.001). Moreover, bodybuilders who smoke are 2.1 times more likely to use steroids (OR = 2.120 [1.407–3.192]; *p* < 0.001).

Table 3 provides the results affecting factors on AAS use for competitive bodybuilders People who are older or have longer time to start bodybuilding is higher are less likely to use AAS (OR = 0.927 [0.641–1.344]; *p* < 0.001, OR = 0.913 [0.847–1.416]; *p* < 0.001). Also, bodybuilders who have higher Physical self-concept have fewer odds to use AAS (OR = 0.798 [0.151–4.225]; *p* < 0.001). For people whose scores of attitude, behavioral intention, and desire are higher, the odds of using AAS increases by 10%, 24%, and 13%, respectively (OR = 1.108 [0.270–4.550]; *p* < 0.001, OR = 1.245 [0.216–7.180]; *p* < 0.001, OR = 1.132 [0.582–2.200]; *p* < 0.001).

**Table 3:** Affecting factors on AAS use for competitive bodybuilders

Variable	$\widehat{{\mathbf{\beta }}}$	SE ($\widehat{{\mathbf{\beta }}}$)	Odds ratio	95% CI	*p*-value
*Intrapersonal factors*					
Age	− 0.075	0.189	0.927	(0.641, 1.344)	< 0.001
Time of starting the bodybuilding	− 0.091	0.131	0.913	(0.847, 1.416)	< 0.001
Body mass index	0.031	0.345	1.032	(0.525, 2.028)	0.194
Attitude	0.102	0.721	1.108	(0.270, 4.550)	< 0.001
Subjective norms	0.016	0.073	1.017	(0.881, 1.172)	0.546
Physical self-concept	− 0.225	0.850	0.798	(0.151, 4.225)	< 0.001
Behavioral intention	0.219	0.894	1.245	(0.216, 7.180)	< 0.001
Behavioral willingness	0.124	0.339	1.132	(0.582, 2.200)	< 0.001
*Interpersonal factors*					
Coach use AAS	0.559	0.138	1.749	(1.334, 2.292)	< 0.001
Friend use AAS	1.039	0.539	2.820	(0.983, 8.129)	< 0.001
*Behavioral factors*					
Alcohol consumption	0.715	0.068	2.045	(1.789, 2.336)	< 0.001
Smoking	0.070	0.249	1.072	(0.658, 1.747)	0.676
Supplement use	0.813	0.252	2.256	(1.376, 3.695)	< 0.001

The odds of using AAS is 70% higher in bodybuilders whose coach also uses AAS (OR = 1.749 [1.334–2.292]; *p* < 0.001). The odds of taking AAS is 2.8 times higher for those whose friend uses AAS (OR = 2.820 [0.983–8.129]; *p* < 0.001). Subjects who use alcohol or supplements have 2, and 2.2 times more likely AAS use (OR = 2.045 [1.789–2.336]; *p* < 0.001, OR = 2.256 [1.376–3.695]; *p* < 0.001, respectively).

Tables 4 and 5 show the result of RMLM for competitive bodybuilders at the national and regional levels. According to Table 4, national-level bodybuilders who are older are less likely to use AAS. Also, at this level of bodybuilders, people who smoke or take supplements are less likely to use AAS (OR = 0.307 [0.148–0.634]; *p* = 0.001, OR = 0.562 [0.328–0.964]; *p* = 0.036, respectively). By increasing BMI, the odds of steroid use increase by 14% (OR = 1.148 [1.019–1.293]; *p* = 0.023).

**Table 4:** Affecting factors on AAS use for national level competitive bodybuilders

Variable	$\widehat{{\mathbf{\beta }}}$	SE ($\widehat{{\mathbf{\beta }}}$)	Odds ratio	95% CI	*p*-value
*Intrapersonal factors*					
Age	− 0.204	0.045	0.815	(0.746,0.891)	< 0.001
Time of starting the bodybuilding	0.018	0.003	1.012	(1.012, 1.025)	< 0.001
Body mass index	0.138	0.061	1.148	(1.019,1.293)	0.023
Attitude	− 0.014	0.066	0.986	(0.865,1.123)	0.829
Subjective norms	0.139	0.115	1.149	(0.917,1.439)	0.227
Physical self-concept	− 0.090	0.054	0.914	(0.822,1.015)	0.093
Behavioral intention	0.040	0.110	1.041	(0.839,1.291)	0.716
Behavioral willingness	0.010	0.080	1.010	(0.864,1.181)	0.899
*Interpersonal factors*					
Coach use AAS	0.933	0.437	2.542	(1.080,5.983)	0.033
Friend use AAS	0.075	0.345	1.078	(0.548,2.120)	0.829
*Behavioral factors*					
Alcohol consumption	0.436	0.390	1.547	(0.720,3.325)	0.264
Smoking	− 1.182	0.370	0.307	(0.148,0.634)	0.001
Supplement use	− 0.576	0.275	0.562	(0.328,0.964)	0.036

**Table 5:** Affecting factors on AAS use for regional level competitive bodybuilders

Variable	$\widehat{{\mathbf{\beta }}}$	SE ($\widehat{{\mathbf{\beta }}}$)	Odds ratio	95% CI	*p*-value
*Intrapersonal factors*					
Age	− 0.049	0.019	0.952	(0.918,0.988)	0.009
Time of starting the bodybuilding	− 0.071	0.002	0.932	(0.928,0.935)	< 0.001
Body mass index	0.023	0.027	1.023	(0.970,1.079)	0.403
Attitude	0.107	0.024	1.113	(1.062,1.165)	< 0.001
Subjective norms	− 0.020	0.032	0.980	(0.921,1.043)	0.523
Physical self-concept	− 0.212	0.024	0.809	(0.771,0.848)	< 0.001
Behavioral intention	0.224	0.039	1.250	(1.159,1.349)	< 0.001
Behavioral willingness	− 0.136	0.041	0.872	(0.805,0.945)	0.001
*Interpersonal factors*					
Coach use AAS	0.332	0.140	1.394	(1.059,1.835)	0.018
Friend use AAS	1.045	0.143	2.842	(2.147,3.762)	< 0.001
*Behavioral factors*					
Alcohol consumption	0.622	0.241	1.862	(1.162,2.985)	0.010
Smoking	0.041	0.185	1.041	(0.725,1.496)	0.826
Supplement use	0.969	0.162	2.636	(1.919,3.621)	< 0.001

Based on Table 5, in competitive bodybuilders at the regional level, older subjects have less likely to use AAS (OR = 0.952 [0.918–0.988]; *p* = 0.009). Also, people who have been doing bodybuilding for a longer period of time are less likely to use AAS (OR = 0.932 [0.928–0.935]; *p* < 0.001). At this level of bodybuilders, when physical self-concept or behavioral willingness is higher, the odds of steroid use are lower (OR = 0.809 [0.771–0.848]; *p* < 0.001, OR = 0.872 [0.805–0.945]; *p* = 0.001, respectively). People who have a higher score in attitude and behavioral intention increase the odds of using AAS by 11% and 25% (OR = 1.113 [1.062–1.165]; *p* < 0.001, OR = 1.250 [1.159–1.349]; *p* < 0.001, respectively).

The coach's use of steroids and alcohol consumption for these bodybuilders increase the odds of using AAS by 39% and 86% (OR = 1.394 [1.059–1.835]; *p* = 0.018, OR = 1.862 [1.162–2.985]; *p* = 0.010, respectively). When a bodybuilder’s friend uses AAS or the bodybuilder takes supplements, the odds of using AAS increases by 2.8, and 2.6 times (OR = 2.842 [2.147–3.762]; *p* < 0.001, OR = 2.636 [1.919–3.621]; *p* < 0.001, respectively).

## Discussion

This study investigated the factors affecting AAS use in non-competitive and competitive bodybuilders. The prevalence rate of AAS use among non-competitive and competitive bodybuilders was 27.5% and 34.1%, respectively,

although the relationship between AAS use and the bodybuilders' status (competitive and non-competitive) was not significant. In the available evidence, more AAS use is seen in both competitive bodybuilders [20] and non-competitive bodybuilders [21]. In our study, about one-third of competitive and non-competitive bodybuilders used AAS. It seems that only competitive goals can't justify the use of AAS. Adolescents' and young adults' attention to body shape and body image through increasing muscle mass has been documented, so that some of these people believe that boys/men with bigger muscles are more attractive [22]. The evidence indicates that the reasons for the use of AAS could be the pressure of the press to compete and win, positive attitude towards doping to achieve success, people's unrealistic expectations from competitions, the pressure of coaches, the lack of monitoring of consumption, the competitive personality of the bodybuilder, attracting the attention of the spectators of sports competitions, the heavy schedule of a bodybuilder's competitions, increasing strength, muscle mass, and endurance pointed out [23–25].

The ridge logistic regression was applied with respect to severe collinearity between predictors. This study showed that younger bodybuilders and those with less experience in the gym were more likely to use steroids in both groups of bodybuilders, which was consistent with Wichstrom [26]. The possible reason for this result could be that with increasing age and more experience using the gym, the intensity of emotions decreases and the person understands the facts more than in the past.

An inverse relationship was found between BMI and AAS use in non-competitive bodybuilders, which confirms the results of the study by Mattila et al. [27]. It is possible that non-competitive bodybuilders attend the gym to build muscles and may use AAS to speed up the process.

The present study revealed a positive association between AAS use with intention and attitude toward AAS use in both groups. Also, AAS use was associated with a lower physical self-concept in both groups. This finding supports in the other studies [28–31]. Positive intention and attitude towards an issue usually lead to doing that behavior, and a negative physical self-concept may cause a person haste in muscle building. For competitive bodybuilders, there was a relationship between behavioral willingness and AAS use. It may be because using steroids is not banned for bodybuilders in competitions hence, they easily show a desire to use.

The use of AAS by coaches and friends has a positive effect on bodybuilders' AAS use. This association was observed in both competitive and non-competitive bodybuilders. These results support previous research findings [32, 33] and can be attributed to the fact that the coach usually gives the bodybuilding exercise program and consultation, and may also sell these steroids. On the other hand, compliments are common in Iranian culture and a bodybuilder may be tempted to receive it by a close friend. Additionally, a friend has the role of a training opponent who is to be beaten by the bodybuilder.

Among behavioral factors, alcohol and supplement use were positively associated with AAS use. This relationship was seen in both groups of bodybuilders. In accordance with the present results, previous studies have demonstrated a positive relationship between alcohol and supplement consumption and AAS use [34–37]. Smoking had a positive effect on steroid use among bodybuilders which the previous study has reported such results [38].

## Limitations

The results of the current study should be interpreted in light of some limitations. First limitation was related to cross-sectional design and observed associations did not show the causality. Another limitation of our study was that data was obtained using self-reported data that may be associated with social desirability bias and due to the risky nature of the behavior of using androgenic-anabolic steroids, some bodybuilders may refuse to tell the truth. Finally, the study was conducted only among male bodybuilders. So, the generalizability of the results is limited because our sample consisted of men.

## Conclusions

The results of the study indicated that a combination of intrapersonal, interpersonal and behavioral factors was effective on the androgenic-anabolic steroids use among Iranian bodybuilders. Adequate education about the side effects of AAS and improvement of individual skills seem to be helpful in reducing AAS use.

## Supplementary Information

**Additional file 1:** Measurement details.

## Data Availability

The analyzed dataset in this study is available from the corresponding author on reasonable request.
