# Supplementary material for: Androgenic-anabolic steroids use among bodybuilders in western Iran: application of ridge logistic regression model
Source: BMC Sports Sci Med Rehabil. 2023 Jan 11;15:7. doi: 10.1186/s13102-023-00616-4 (PMC9835229; doi:10.1186/s13102-023-00616-4)
Supplement: Supplementary file 1 — Additional file 1: Measurement details. [file 13102_2023_616_MOESM1_ESM.docx]

**Measurement**

A self-administrated questionnaire was used. Face and content validity of the questionnaire was assessed by ten experts in health education and promotion. We calculated content validity ratio (CVR) and content validity index (CVI). The values of 0.62 for CVR and 0.79 for CVI. In order to measure the reliability of the questionnaire, a pilot study was conducted with a sample size of 30 bodybuilders, and the alpha coefficient was calculated. This questionnaire consisted of three sections: intrapersonal, interpersonal, and behavioral factors.

Intrapersonal factors: intrapersonal factors included the following factors.

Attitude toward androgenic-anabolic steroids use was measured by six items (e.g., “In my opinion, androgenic-anabolic steroids can cause dangerous diseases”.) on a 5- point Likert scale (1= strongly disagree, 5= strongly disagree). The range of attitude score was 6-30. A higher score meant more positive attitude towards using androgenic-anabolic steroids. Cronbach's Alpha coefficient was 0.79.

Subjective norms about using androgenic-anabolic steroids were measured by six items (e.g., “If I use androgenic-anabolic steroids, my coach will approve it”.) on a 5- point Likert scale (1= not at all, 5= very much). The range of subjective norms score was 6-30. A higher score meant encourager subjective norms about using androgenic-anabolic steroids. Cronbach's Alpha coefficient was 0.70.

Physical self-concept about using androgenic-anabolic steroids was measured by seven items (e.g., “I am a physically strong person”.) on a 5- point Likert scale (1= strongly disagree, 5= strongly disagree). The range of Physical self-concept score was 7-35. Cronbach's Alpha coefficient was 0.73.

Behavioral intention about using androgenic-anabolic steroids was measured by four items (e.g., “I would like to use androgenic-anabolic steroids within the next month to improve my athletic performance”.) on a 5- point Likert scale (1= not at all, 5= very much). The range of behavioral intention score was 6-30. In this part, the person's intention to use anabolic steroids now, one month and the next six months is considered.

Behavioral willingness about using androgenic-anabolic steroids was measured by ten items (e.g., “My friend who regularly uses androgenic-anabolic steroids is strong”) on a 5- point Likert scale (1= not at all, 5= very much). The range of behavioral willingness score was 10-50. A higher score meant the individual's positive behavioral willingness of his peers who use. Cronbach's Alpha coefficient was 0.79. Age, time of starting bodybuilding, and body mass index (BMI) were other intrapersonal factors measured.

Interpersonal factors: Interpersonal factors contained AAS use by coaches and friends.

Behavioral factors: Behavioral factors included alcohol consumption, smoking, and supplement use.

The questionnaire has been attached as follows.

**In the name of God**

**Dear participant,**

The present questionnaire aims to determine factors affecting the androgenic-anabolic steroids use in Iranian bodybuilders. It takes approximately 15 minutes to complete this questionnaire. Your participation in this study is entirely voluntary. The information and data are strictly confidential. We will publish only aggregated, anonymous results.

Note: The study was approved by the Ethics committee of Hamadan University of Medical Sciences, Iran (No. IR.UMSHA.REC.1395.356).

Thank you for your support.

**Signature……………………………… Date………………………….**

**Answer Guide:** Please, choose the most suitable option in your opinion.

| A**ge (year): ………... Weight: ………** kg **Height:** ………. cm | | | | | |  |
| --- | --- | --- | --- | --- | --- | --- |
| **Time of starting the bodybuilding: …………………………...** month(s**)** | | | | | |  |
| **Marital status**  Married  Single | | | | | |  |
| **Education level**  Under diploma  Diploma  Academic | | | | | |  |
| **Occupation**  Employed  Self-employed  Unemployed | | | | | |  |
| **AAS use**  Yes  No | | | | | |  |
| **Coach use AAS**  Yes  No | | | | | |  |
| **Friend use AAS**  Yes  No | | | | | |  |
| **Alcohol consumption**  Yes  No | | | | | |  |
| **Smoking**  Yes  No | | | | | |  |
| **Supplement use**  Yes  No | | | | | |  |
| **Type of bodybuilding**  Competetive  Non-competitive | | | | | |  |
| **If you work Competetive bodybuilding, what is your level competitive?**  National level  Regional level | | | | | |  |
| **Attitude** | Strongly disagree | Disagree | Neutral | Agree | Strongly agree | |
| 1) Androgenic-anabolic steroids help me have a stronger body. |  |  |  |  |  | |
| 2) Taking androgenic-anabolic steroids helps to perform better in competitions. |  |  |  |  |  | |
| 3) In my opinion, androgenic-anabolic steroids can cause dangerous diseases. |  |  |  |  |  | |
| 4) Taking androgenic-anabolic steroids is a good way to build and grow muscles. |  |  |  |  |  | |
| 5) Androgenic-anabolic steroids help me to attract the attention of others. |  |  |  |  |  | |
| 6) Taking androgenic-anabolic steroids helps me reach my athletic goals more easily. |  |  |  |  |  | |
| **Subjective norms** | Not at all | Not really | Neutral | Somewhat | Very much | |
| 1) It is possible to access and easily obtain androgenic-anabolic steroids for me. |  |  |  |  |  | |
| 2) A lot of people in your club use androgenic-anabolic steroids. |  |  |  |  |  | |
| 3) If I use androgenic-anabolic steroids, my coach will approve it. |  |  |  |  |  | |
| 4) If I use androgenic-anabolic steroids, my teammates will approve. |  |  |  |  |  | |
| 5) If I use androgenic-anabolic steroids, my best friend will approve. |  |  |  |  |  | |
| 6) I follow the opinions of others regarding androgenic-anabolic steroids. |  |  |  |  |  | |
| **Physical self-concept** | Strongly disagree | Disagree | Neutral | Agree | Strongly agree | |
| 1) I have too much fat in my body. |  |  |  |  |  | |
| 2) I am satisfied with myself physically. |  |  |  |  |  | |
| 3) I am a physically strong person. |  |  |  |  |  | |
| 4) I perform coordinated movements with confidence. |  |  |  |  |  | |
| 5) I have good sports skills. |  |  |  |  |  | |
| 6) My body is soft and flexible |  |  |  |  |  | |
| 7) I can do physical activity for a long time without getting tired. |  |  |  |  |  | |
| **Behavioral intention** | Not at all | Not really | Neutral | Somewhat | Very much | |
| 1) I intend to use androgenic-anabolic steroids in the next 6 months to improve my athletic performance. |  |  |  |  |  | |
| 2) I would like to use androgenic-anabolic steroids within the next month to improve my athletic performance. |  |  |  |  |  | |
| 3) I would recommend androgenic-anabolic steroids to my friends. |  |  |  |  |  | |
| 4) I plan to continue bodybuilding only through exercise and without the use of androgenic-anabolic steroids. |  |  |  |  |  | |
| **Behavioral willingness** | Not at all | Not really | Neutral | Somewhat | Very much | |
| 1) I imagine one of my bodybuilder friends who regularly uses androgenic-anabolic steroids is attractive. |  |  |  |  |  | |
| 2) I imagine one of my bodybuilder friends who regularly uses androgenic-anabolic steroids is happy. |  |  |  |  |  | |
| 3) I imagine one of my bodybuilder friends who regularly uses androgenic-anabolic steroids is proud. |  |  |  |  |  | |
| 4) I imagine one of my bodybuilder friends who regularly uses androgenic-anabolic steroids is sincerely. |  |  |  |  |  | |
| 5) I imagine one of my bodybuilder friends who regularly uses androgenic-anabolic steroids is strong. |  |  |  |  |  | |
| 6) I imagine one of my bodybuilder friends who regularly uses androgenic-anabolic steroids is nervous. |  |  |  |  |  | |
| 7) I imagine one of my bodybuilder friends who regularly uses androgenic-anabolic steroids has high sexual desire. |  |  |  |  |  | |
| 8) I imagine one of my bodybuilder friends who regularly uses androgenic-anabolic steroids is bully. |  |  |  |  |  | |
| 9) I imagine one of my bodybuilder friends who regularly uses androgenic-anabolic steroids eagers to use steroids. |  |  |  |  |  | |
| 10) I imagine one of my bodybuilder friends who regularly uses androgenic-anabolic steroids is illiterate. |  |  |  |  |  | |

***Thank you for your participation.***
